# Exploring School Staff's Perceptions About Implementing Saliva‐Based Testing

**DOI:** 10.1002/puh2.225

**Published:** 2024-07-24

**Authors:** W. C. Cheung, M. M. Ostrosky, C. O'Grady, M. Chudzik, A. Ackerman, N. Perez, N. Delinski, R. L. Smith

**Affiliations:** ^1^ College of Health and Human Sciences, Allied Health and Communicative Disorders Northern Illinois University DeKalb Illinois USA; ^2^ College of Education, Special Education University of Illinois Urbana‐Champaign Urbana Illinois USA; ^3^ College of Liberal Arts and Sciences, Anthropology University of Illinois Urbana‐Champaign Urbana Illinois USA; ^4^ Carle-Illinois College of Medicine, Biomedical and Translational Sciences University of Illinois Urbana‐Champaign Urbana Illinois USA; ^5^ College of Education, Early Childhood Special Education University of Alabama Tuscaloosa Alabama USA; ^6^ Loyola Marymount University Los Angeles California USA; ^7^ Jump Trading Simulation and Education Center Peoria Illinois USA

**Keywords:** COVID‐19, K‐12 schools, saliva‐based testing, school staff, teachers

## Abstract

**Background:**

To investigate the feasibility of conducting COVID‐19 testing in kindergarten‐grade 12 schools, saliva‐based testing was implemented in five schools. The purpose of this study was to understand staff's perspectives of the barriers and facilitators to conducting saliva‐based testing in their settings.

**Methods:**

Thirty‐three school staff who worked in five target schools were interviewed in the summer of 2021. Participants were recruited from June–August 2021 via a flyer that was distributed through email and school‐affiliated social media and websites, and word‐of‐mouth. Semi‐structured interviews were conducted over Zoom by a trained interviewer; interviews were audio recorded and auto transcribed using this Zoom feature. Constant comparative analysis and emergent coding were used to analyze the data.

**Results:**

The majority of participants had positive experiences with conducting saliva‐based testing during school hours. Participants reported that saliva‐based testing did not interrupt their school routine because (1) the testing was simple and quick, (2) the testing schedule was consistent and organized, and (3) school staff maintained open lines of communication. Barriers to implementing the testing were as follows: (1) obtaining parental consent, (2) not being allowed to drink or eat an hour before testing, and (3) struggling to provide enough saliva for testing. Participants suggested the following strategies to facilitate testing in schools: being more proactive, giving families fewer things to sign (i.e., consent forms), improving communication, adding needed consents to the school registration process, using social media, and increasing community outreach.

**Conclusion:**

As schools weigh the benefits and the risks of closing for extended periods of time versus remaining open for in‐person learning, saliva‐based testing is a feasible and efficient way to support programs in this decision‐making process. This approach can be used in future pandemics and in areas with outbreaks or poor vaccine coverage.

## Graphical abstract

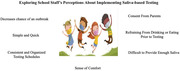

## 1 Introduction

The COVID‐19 pandemic led to many school closings, resulting in long‐term economic and educational costs. The Brookings Institution [3, 4] reported that 4 weeks of school closures in New York City cost $1.1 billion, and a nationwide closure for 12 weeks cost 1% of the gross domestic product (GDP). Research showed that academic loss disproportionately impacted students from families with limited resources [5, 6, 7]. For example, school closures resulted in an average of 5–9 months learning loss in mathematics by students [5, 6, 7]. Students from low‐income families were disproportionately impacted with estimates showing a 6–12‐month loss in mathematics learning [6]. It is important to find ways to keep schools open under circumstances such as pandemics and inclement weather, particularly schools in underserved and low‐income communities. Give the importance of keeping schools open during the pandemic, Federal Executive Order 14000 was issued on January 26, 2021, which emphasized that K‐12 schools must be supported in opening and continuing to operate to minimize the academic impact on students.

Although almost all schools resumed in‐person learning with control measures in May 2021, and most schools now offer in‐person learning without any control measures, exploring strategies to prepare against future pandemics and similar extreme situations so that schools remain open is necessary. The control measures that were used in 2021, during the COVID‐19 pandemic (i.e., the period when this study was conducted), included masking, social distancing, improved ventilation, and creating separate lunch periods. These were important measures and helped minimize the risk of transmission. However, research [11] demonstrated that increased testing enabled schools to continue operating. Such testing allowed schools to implement contact tracing and isolate individuals who tested positive or had symptoms, thereby providing a safer learning environment for all students. Although increased testing can help avert a future epidemic wave [9], the ability to perform large‐scale testing in schools has remained elusive [12].

Saliva‐based testing has been shown to be more sensitive than nasopharyngeal swab testing [13]. It has the potential for large‐scale testing in schools [10] because (1) samples can be collected by school staff rather than healthcare workers, (2) it is noninvasive that may be less stressful for students, and (3) it does not require swabs and other medical items that can create supply chain issues [14]. Research has revealed that garnering staff buy‐in and participation are key to performing large‐scale testing in schools [14]. However, there is no research that has explored the perceptions of school staff about their experiences of *having saliva‐based testing in their schools*.

This study was part of a larger study that examined the feasibility of implementing saliva‐based testing in K‐12 schools in underserved communities. Five Illinois schools participated in this larger study, including one high school (9th–12th grades), one elementary school (3th–5th grades), one middle school (6th–8th grades), and two combined schools (K‐8th and 3rd–8th). Two of these schools were in suburban areas, two were in urban settings, and one was situated in a rural area. Because of the different needs of students at each grade level, the five schools were selected to cover every grade level. All these schools were located in under resourced and vulnerable communities, and in each school more than 80% of students were eligible for free or reduced (priced) lunch. Saliva‐based testing was provided to these schools at no cost; it required formal consent for students under 18 years old to participate. Because of the diverse population in these schools, consent forms were provided in both English and Spanish. This large study was conducted in five schools from January to May in 2021; schools began to shift from in‐person teaching with restrictions to completely reopening without restrictions during these 5 months. The goal of the larger study was to train school personnel on how to set up saliva collection sites, effectively collect samples, and resolve logistical issues within their schools. The logistics on conducting saliva‐based testing in schools are not discussed in this paper, rather the focus is on staff experiences.

According to Protection Motivation Theory [19], people are motivated to participate in events when they believe that the method will be easy and effective. To successfully implement saliva‐based testing schoolwide, staff must be motivated and willing to participate in it. In the larger study that was the backdrop for this data set, there was a lot of communication between school staff and families. Given that participation in testing was voluntary, how messages were delivered could have impacted parents’ decisions regarding their children's participation.

Realizing that pandemics are part of the world's landscape and that future pandemics may require school‐based testing, it is important to understand school staff's perspectives about saliva‐based testing. The purpose of this study was to explore the perceptions of school staff who worked in five schools where saliva‐based testing was freely available. This study was guided by three questions: (1) How do school staff describe their experiences with saliva‐based testing in their schools? (2) What do school staff see as facilitators and barriers to implementing saliva‐based testing? and (3) What strategies do school staff believe would help facilitate saliva‐based testing?

## 2 Methods

### 2.1 Participants

Thirty‐three school staff participated in this interview study. School staff were defined as anyone who worked in the target schools such as teachers, therapists, administrators, secretaries, and janitors. Therefore, our only inclusion criterion was that participants had to work in one of five target schools in Illinois during January–May in 2021, though they were not required to participate in saliva‐based testing themselves. More than half of the participants were teachers. Most participants were between 25 and 35 years of age and the majority of them were female and White. Almost all participants reported that they had earned college degrees, and the majority of them worked in public schools. Furthermore, most participants had been in their current position for 5 years or less (see Table 1).

**TABLE 1:** Participant demographics (*N* = 33).

Demographic	*n*	%
Gender		
Female	27	81
Male	6	19
Age (years)		
Younger than 25	1	3
25–35	15	45
36–45	6	18
46–55	7	21
Older than 55	4	12
Race/Ethnicity		
White	23	70
Black/African American	3	9
Hispanic/Latino	3	9
Multiracial	2	6
Marital status		
Married	18	55
Not married	14	42
No response	1	3
Highest level of education		
Some college	1	3
Bachelor's degree	9	27
Master's degree	21	63
Doctorate	1	3
School setting		
Public	28	85
Private	4	12
No response	1	3
Position		
General education teacher	17	52
Special education teacher	2	6
Administrator	7	21
Other	6	18
No response	1	3
Years in position		
5 or less	20	61
6–10	6	18
11–20	4	12
21 or more	2	6
No response	1	3

### 2.2 Recruitment

Participants were recruited from June to August 2021 via a flyer that was distributed through email and school‐affiliated social media and websites, or word‐of‐mouth (snowball sampling). Recruitment was ongoing until redundancy was noted in the themes [1]. Each participant received a $40 gift card for their participation, in appreciation for their time. Specifically, each participant received a $20 gift card after the interview and a $20 gift card after the member check.

### 2.3 Procedures

This study was ruled exempt by the University of Illinois Institutional Review Board (IRB#21961). If an individual expressed an interest in participating in the study and met the inclusion criteria, they received a consent form, demographic questionnaire, and scheduled an interview. Semi‐structured interviews were conducted in the summer of 2021 via Zoom by trained interviewers; interviews were audio recorded and auto transcribed using the transcription feature on Zoom. Notably, Zoom is HIPPA compliant [26]. Extant literature has suggested that in‐person, phone, and Zoom interviews may generate similar results [27, 28]. Two interviewers were female; they did not know the study participants. On average, interviews lasted 38 min (ranging from 21 to 62 min). After each interview, the interviewer emailed an interview summary to each participant to ensure the accuracy of the content (i.e., member check). No participant made changes to their summary.

### 2.4 Instruments

The interview protocol was developed on the basis of existing literature [24] and reviewed by experts in educational research and public health research. Both the demographic questionnaire and interview protocol were pilot tested with a teacher whose data were not included in the study. As a result of the pilot, changes were made to the protocol (e.g., minor wording changes to help with flow and clarity of questions), and the interview protocol was finalized.

The questionnaire included demographic information about each participant as well as information related to their job, including their highest level of education, type of school in which they taught (public or private), position, and years in their current position. The questionnaire was developed by the research team and reviewed by several faculties with experience in public policy, civil engineering, and nursing. English and Spanish versions of the questionnaire were available.

### 2.5 Data Analysis

One member of the research team listened to each interview and corrected the transcripts as needed. Two team members read each transcript multiple times to familiarize themselves with the data [20]. Constant comparative analysis [21] and emergent coding [22] were used to code the transcripts. The two team members independently coded the interviews, using a line‐by‐line approach. Their coded data were compared and annotated with specific phrases [23]. Each new piece of data was then compared with previously coded data to determine if it represented a novel idea or matched an existing code. A codebook was developed at this stage; it included codes, definitions, and examples. The researchers met weekly to compare codes and resolve differences. Then, they used the codebook to reexamine the data. During this process, they reviewed each other's codes and reached consensus, with oversight from the first two authors throughout the coding process. Some questions or data groups were coded by themes or patterns that emerged, whereas others were coded by tallying responses. For example, for the question “When saliva‐based testing was implemented at your school, what did you find helpful about the process, or what worked well about the process?” one sample code was: the organization of the testing process and staffing. For questions such as “Do you think it's important for students to take saliva‐based testing at your school?,” the responses of “yes,” “no,” and “unsure” were counted. The coding process was conducted over approximately 2 months.

After all data were coded, the codes were grouped into categories and organized into themes grounded in the data. For example, the theme about positive experience was derived from the following two categories: sense of comfort and decrease chance of outbreak. When coding disagreements arose, team members discussed the codes until consensus was reached. Prior to data analysis, participants’ names were replaced with pseudonyms and identifiable information was removed from summary documents.

### 2.6 Researcher Identity

The research team included two special education PhDs, four students from special education and anthropology, and one epidemiologist. All team members had experience working with teachers and understood school systems and health issues. Finally, all team members had participated in saliva‐based testing and were familiar with the process. The researchers’ identities shaped the lens to which they approached the study as several team members focused on medical and societal implications, whereas others focused on educational considerations and implications.

### 2.7 Trustworthiness

We made several efforts to ensure the credibility and trustworthiness of the data and the themes. For example, a member check was conducted after each interview by the participant. For the member check, a 1–2‐page summary of the interview was written by a research team member and emailed to the participant for feedback. All participants responded to the member check; however, no participant made changes to their summary. By conducting member checks, participants were able to assess the validity of the findings [8]. In addition to the member check, the research team members debriefed with one another throughout data collection and data analysis. Further, team members met weekly to refine themes during data analysis [2].

### 2.8 Ethical Considerations

This study was ruled exempt by the University of Illinois Urbana‐Champaign (UIUC) Institutional Review Board (IRB #21961). Participants were voluntary, and consents were obtained.

## 3 Results

Four themes were identified related to the research questions (positive experiences, facilitators to saliva‐based testing, barriers to saliva‐based testing, and strategies to facilitate testing). Each theme is discussed in the following sections along with sample participant quotes.

### 3.1 Positive Experiences

The majority of participants had positive experiences with saliva‐based testing. They shared that saliva‐based testing and mask wearing were the easiest strategies to implement, whereas maintaining social distancing was the most difficult. Fifty‐three percent of the participants rated saliva‐based testing as extremely important compared with other strategies. When discussing their positive experiences, participants noted that saliva‐testing increased their sense of comfort and decreased their worries about the chance of an outbreak in their setting.

#### 3.1.1 Sense of Comfort

Participants reported that having access to saliva‐based testing provided a sense of comfort to parents and staff and decreased the chance of an outbreak. For instance, JinHee shared, “There is the actual scientific usefulness of getting the information and using it to keep children safe. And I think there is the psychological usefulness of creating a school environment that feels safe to students, families, and to staff.” Rachel similarly shared, “I think it was extremely important … it just gave me that peace of mind going into the building if there was someone who had COVID‐19, they were most likely sent home to quarantine. I just felt like the test was clear … it made me feel confident.” Similarly, Jean mentioned that the fast results gave her peace of mind: “It gave everybody a sense of comfort, so we did have students or staff that would test positive … but we were able to act quickly and do contact tracing.” In addition to providing school personnel with a sense of security at work, saliva‐based testing also helped families feel more comfortable allowing their children to return to in‐person learning. For example, Amy said, “Some families who opted out, and then when we started doing the saliva‐based testing, they asked if their student could join in … and come into the school, so I do think that brought some ease to the minds of the parents.”

#### 3.1.2 Decreases Chance of an Outbreak

In addition to an increased sense of comfort, participants shared that saliva‐based testing was an effective strategy for limiting the chance of an outbreak. For example, Lauren shared: “I think it greatly reduced the spread of COVID‐19 in our school and community because as soon as those kids tested positive, they immediately took care of making sure everyone else was safe.” Similarly, participants felt that the availability of saliva‐based testing had a positive impact on their school remaining open for in‐person learning. For example, Edward expressed: “It would lower the chance of an outbreak and it would lower the chances of having the whole classroom need to stay home … it just reduces the unnecessary quarantining of people … I feel like in the beginning they were sending everybody home, like whole classrooms, without knowing if people had it, so this would reduce that from happening.”

### 3.2 Facilitators to Saliva‐Based Testing

When participants were asked why saliva‐based testing was one of the easiest strategies to implement in schools, they reported that (1) it was simple and quick to do, (2) the testing schedule was consistent and organized, and (3) school staff maintained open lines of communication.

#### 3.2.1 Simple and Quick

Many participants reported that saliva‐based testing was simple and quick to do. For example, Jose stated, “In terms of the actual test, it was very simple to do, and even our kindergartners could do it so that was helpful … it didn't take a lot of class time.” Similarly, Edward said, “It was such a simple thing to do. You get to spit in it, and you just drop it [put vial into a container]. It was so simplistic. It was easy to follow.”

#### 3.2.2 Consistent and Organized Testing Schedules

Most participants noted that the organized and consistent testing schedule did not interrupt their instructional time and routines. For instance, Kate shared, “… the schedule and the planning made it really easy … the test itself was so fast that it was easy for the kids to do it. And it also didn't take away from the rest of the stuff we needed to do during the day.” Similarly, May stated, “The administration really organized what time students were going to come down, and they put the students in groups … so that was very organized and worked very well.” Likewise, Jamie shared, “So the process for a teacher was pretty seamless. There were a multitude of volunteers, and our assigned time was Tuesdays and Thursdays at 10am … so the same volunteer would come to the classroom … they would call the names of the students that had signed up … I didn't have to change any of my routines or practices or anything.”

#### 3.2.3 Communication

Most participants also mentioned that good communication within their school was critical to the success of saliva‐testing. For example, Karen shared, “Our principal made a great video on how to do the testing and that was really helpful because he walked [us] through the whole process and so it was nice to see ‘this is what you do’.”

### 3.3 Barriers to Saliva‐Based Testing

Participants reported that the major difficulties to implementing saliva‐based testing were (1) obtaining parental consent, (2) not allowing children to drink or eat for an hour prior to testing, and (3) children struggling to provide enough saliva.

#### 3.3.1 Consent From Parents

Most participants reported that getting parents to consent to testing was difficult. Caroline said, “Just getting people to sign up, getting people to see the importance of getting this testing done in school is difficult.” Many participants shared that the major barriers to obtaining parents’ consent included privacy concerns, misunderstanding the testing procedures, language and literacy issues, and parent perspectives toward COVID‐19. For example, Jenny shared that one family “… wanted to know if a positive test occurred, where that information would be shared … who we were reporting it to, how [we were] reporting it, just protecting the family's privacy.” Some participants also noted that some families did not understand the testing procedures, as one person shared, “I think that there were probably folks who had to do the deep, the brain tickling nasal swab and didn't want their students to go through that twice a week, every week.”

Language and literacy concerns also were mentioned when written information, including consent forms, was shared. For instance, Marybeth explained, “Even if you have things translated into Spanish and French, many of them are illiterate in any language. And so, you have a Spanish flyer, that doesn't help … because some of our Spanish speaking families, they can't read it in Spanish either.” Finally, participants shared their struggles with parents who were skeptical of the pandemic in general. As one participant stated, “I don't think most of the parents were taking COVID‐19 very seriously and believe it is a thing.”

#### 3.3.2 Refraining from Drinking or Eating Prior to Testing

Participants noted that for some individuals the testing requirements were difficult to follow, resulting in a barrier to saliva‐testing. For example, Helen said, “The hardest one is not being able to eat or drink or chew gum for an hour. I think it was very hard for middle schoolers.” Similarly, Rachel said, “The hardest part for me was just to remember not to eat or brush my teeth at certain times an hour beforehand … I think there were two times that I forgot. So, then I had to do it [testing] a different day. But for me being so busy … I didn't really have time to eat afterwards.”

#### 3.3.3 Difficult to Provide Enough Saliva

Many participants also noted that the process of filling the vial with enough saliva was difficult. For example, Jessica stated, “I didn't know I have to give so much spit, my mouth wasn't wet enough and it was just hard.” Likewise, Cathy mentioned, “I was aware of the kids were motivated to do it, and they got it done. And others just like not being able to make enough saliva and they eventually were able to do it just fine with practice.”

### 3.4 Strategies to Facilitate Testing

When asked for suggestions to assist with testing at school, most participants focused on strategies to improve the return rate of consent forms. Suggestions included being more proactive, giving families less documents to sign, improving communication, adding needed consents to the school registration process, using social media, and increasing community outreach. For example, Sue offered these ideas: “Social media has been helpful for us this year, to be able to connect quickly. But I think the biggest difference is to bring information to the families and to the communities and stop expecting families and communities to come to us … just keeping things simple and even if it's an app where you just click the button instead of filling out a lot of things or having a lot of things to read. You try to make communication quick like Google Forms and something you can open up on your phone, something you don't have to print, that is a barrier, as well.”

## 4 Discussion

The findings from this exploration of school staff's perceptions regarding saliva‐based testing resulted in several topics that are worthy of discussion. First, most participants held positive views about implementing saliva‐based testing in schools, which is consistent with previous studies [15, 16]. School staff believed that frequent testing helps everyone in the school feel safe [16], which is important for them, so they did not put their families’ health in danger as they returned to in‐person schooling. In addition, school staff were supportive of saliva‐based testing because the process was quick and easy, a finding echoed by other researchers [11]. Research has shown that educators’ acceptability of COVID‐19 testing is influenced by the type of specimen collection offered. Researchers [11] found that more than one‐third of educators and students who agreed to saliva‐based testing reported that they would have declined testing if nasal swabs were used instead. Many individuals feel that saliva‐based testing is less invasive than other methods. Moreover, educators and students are less anxious and less stressed with this testing method [25] and prone to be more positive and agreeable to participate in this format of testing. Therefore, educators’ attitudes and motivation may be a key determinant of the success of saliva‐based testing in schools.

Second, efforts to establish testing schedules and engage in frequent and open communication with school staff were important aspects in making saliva‐based testing feasible. This is consistent with the previous research which demonstrated that clear communication about testing procedures delivered by trusted leaders is necessary to help school communities understand the strengths and limitations of the program and encourage participation [24]. The participants in the current study felt empowered and supported when they saw their school administrator in a video demonstrating the testing procedures. Communication between school leaders and educators is important to facilitate testing programs such as the one highlighted in this study.

One major barrier to implementing saliva‐based testing that was noted by participants was the fact that food and drinks were banned for 45–60 min prior to testing. This can be especially difficult for students. A majority of students ate breakfast provided by their schools, so they had to participate in saliva‐based testing during instructional hours, thereby interrupting class routines and possibly access to academic content. In addition, teachers found it difficult to make sure that younger students did not drink water prior to testing for students often have their own water bottles and are encouraged to stay hydrated. For middle or high schoolers, participants found it difficult to keep students from chewing gum prior to testing. Researchers may need to assess the feasibility of adapting testing procedures for K‐12 students and educators.

Finally, a major barrier to implementing saliva‐based testing was obtaining parent consent. Participants believed that mistrust and misunderstanding the testing procedures were the main obstacles. These issues often were related to communication between schools and parents [24]. In terms of testing procedures, previous studies revealed that nearly 60% of parents or guardians indicated that they would not agree to free testing that required nasal swab specimens [7]. In terms of obtaining consent, school leaders may need to proactively use their school registration process as a setting to explain school‐based testing procedures such as those used to identify the COVID‐19 virus. In particular for multicultural families, it is critical to use face‐to‐face interactions as contexts to develop rapport and build trust between school personnel and family members. Thus, communication between school administrators and educators as well as between school staff and parents is important to overcome logistical issues and to increase participation in school‐based testing programs.

## 5 Directions for Future Research

Further research should focus on students’ perceptions of saliva‐based testing and determine strategies to overcome these barriers. It is important to explore students’ experiences and their perceptions of taking saliva‐based testing in their schools. Findings from the current study suggest that some barriers impacted students’ access or success with saliva‐based testing such as the prohibition on food or drink for 45–60 min prior to testing. There may be additional barriers that impact students’ motivation to participate in schoolwide testing that may not be identified from an educator's perspective. There is also a need to develop a more practical testing protocol such as decreasing the amount of time without food or drinks or decreasing the amount of saliva needed for testing. Further, this study suggests multiple ways to simplify the consent process that might result in an increased return rate. Researchers should work to identify such variables so that the number of students who participate in testing can be improved.

## 6 Implications for School Health Policies and Practice

As closing schools for extended periods of time has negative psychosocial, societal, and economic impacts on children and their families [17, 18], keeping students safe and schools open is an important international priority. Findings from this study suggest that barriers exist which impact students’ and their families’ participation in saliva‐based testing. There may be other barriers that impact students’ participation in saliva‐based testing that were not identified by staff in the current study. However, this approach is generally acceptable from a staff perspective.

## 7 Limitations

Our findings should be interpreted with caution due to several limitations. First, this was a convenient sample that included schools with a high percentage of children who received free or reduced lunch. Staff volunteered to participate, and they and their students had access to saliva‐based testing free of charge. Thus, the findings might not generalize to other school populations. Second, data were collected in summer 2021, when the prevalence of COVID‐19 was high, vaccines were not yet available for young children, and the targeted schools had not yet returned fully to in‐person schooling. All schools that participated in data collection were in Illinois, which had implemented mandatory in‐school masking and state‐funded optional in‐school testing (via saliva or nasal swab, based on school preference) throughout the study period. Attitudes toward saliva‐based testing may have changed as the data were collected, especially as control policies changed. Future research should identify students’ perceptions toward saliva‐based testing as there are still some students who have not been vaccinated due to their medical status [29], family beliefs [30], access [31], and so on. Third, each participant was only interviewed once; their experiences with saliva‐based testing may have shifted over time.

## 8 Conclusion

As schools weigh the benefits and the risks of closing for extended periods of time due to factors such as the COVID‐19 pandemic, versus remaining open for in‐person learning, saliva‐based testing is a feasible and efficient way to support programs in this decision‐making process. This approach can be used in future pandemics and in areas with outbreaks or poor vaccine coverage.

## Author Contributions

**W. C. Cheung:** conceptualization, methodology, formal analysis, supervision, funding acquisition, writing—original draft, writing—review and editing, project administration, and resources. **M. M. Ostrosky:** conceptualization, methodology, formal analysis, supervision, writing—review and editing, writing—original draft, funding acquisition. **C. O'Grady** and **M. Chudzik:** formal analysis, writing—review and editing, writing—original draft. **A. Ackerman** and **N. Perez:** methodology, data curation, writing—review and editing. **N. Delinski:** conceptualization, funding acquisition, methodology, writing—review and editing. **R. L. Smith:** conceptualization, methodology, funding acquisition, writing—review and editing.

## Ethics Statement

The data presented here have not been published elsewhere, and all research activities were approved by the University of Illinois Urbana‐Champaign (UIUC) Institutional Review Board (IRB #21961).

## Conflicts of Interest

The authors declare no conflicts of interest.

## Data Availability

The data supporting this study's findings are available from the first author upon reasonable request.
